# Author Correction: Cross-phyla protein annotation by structural prediction and alignment

**DOI:** 10.1186/s13059-024-03196-9

**Published:** 2024-02-19

**Authors:** Fabian Ruperti, Nikolaos Papadopoulos, Jacob M. Musser, Milot Mirdita, Martin Steinegger, Detlev Arendt

**Affiliations:** 1 Developmental Biology Unit, European Molecular Biology Laboratory, Heidelberg, Germany; 2 Faculty of Biosciences, Collaboration for Joint Ph.D. Degree between EMBL and Heidelberg University, Heidelberg, Germany; 3 Department for Evolutionary Biology, University of Vienna, Vienna, Austria; 4 School of Biological Sciences, Seoul National University, Seoul, South Korea; 5 Artificial Intelligence Institute, Seoul National University, Seoul, South Korea; 6 Centre for Organismal Studies, University of Heidelberg, Heidelberg, Germany

**Author Correction: Genome Biol 24, 113 (2023)**

**https://doi.org/10.1186/s13059-023-02942-9**

Following publication of the original article [1], the authors identified two errors in the citations.

First, in citation 109, the predicted *S. lacustris* protein structures are not deposited under 10.5452/ma-yoep2 but rather 10.5452/ma-coffe-slac.

Second, the Biological Structure Model Archive was cited incorrectly in place of the ModelArchive; citation 11 should therefore point to:

Schwede T, Sali A, Honig B, Levitt M, Berman HM, Jones D, Brenner SE, Burley SK, Das R, Dokholyan NV, Dunbrack RL Jr, Fidelis K, Fiser A, Godzik A, Huang YJ, Humblet C, Jacobson MP, Joachimiak A, Krystek SR Jr, Kortemme T, Kryshtafovych A, Montelione GT, Moult J, Murray D, Sanchez R, Sosnick TR, Standley DM, Stouch T, Vajda S, Vasquez M, Westbrook JD, Wilson IA. Outcome of a workshop on applications of protein models in biomedical research. Structure. 2009 Feb 13;17(2):151–9. 10.1016/j.str.2008.12.014. PMID: 19217386; PMCID: PMC2739730.
