# Supplementary material for: Cross-phyla protein annotation by structural prediction and alignment
Source: Genome Biol. 2023 May 12;24:113. doi: 10.1186/s13059-023-02942-9 (PMC10176882; doi:10.1186/s13059-023-02942-9)
Supplement: Supplementary file 1 — Additional file 1. Supplementary material [112–125]. [file 13059_2023_2942_MOESM1_ESM.pdf]

SUPPLEMENTAL MATERIAL

Cross-phyla protein annotation by structural prediction and alignment

Fabian Ruperti
, Nikolaos Papadopoulos
, Jacob M. Musser
, Milot Mirdita
, Martin Steinegger
and Detlev Arendt

Appendix A: MorF overview

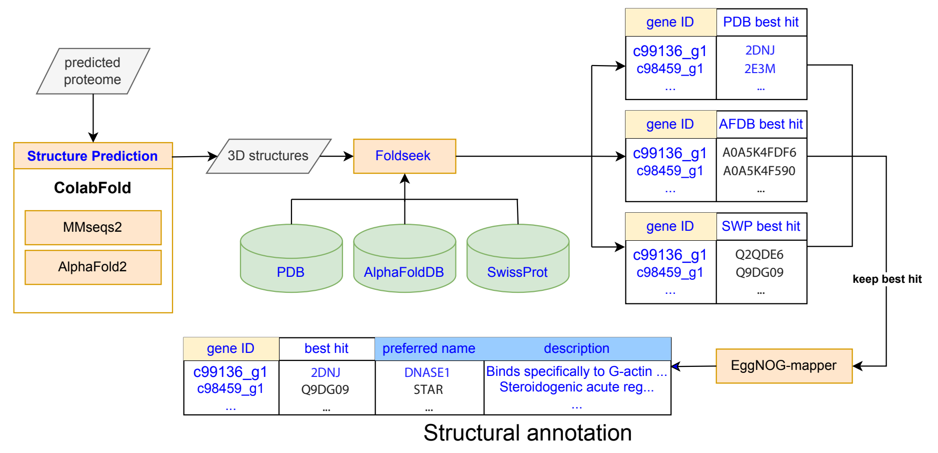

Figure S1 Overview of the MorF workflow ColabFold is called with a proteome as input. It produces multiple sequence alignments for each protein using MMseqs2 and predicts structures for each structure using AlphaFold2. The best model per protein is kept and used as query in Foldseek to search against PDB, AlphaFoldDB, and SwissProt. This creates an m8-formatted table for each database, where the significant hits for each query protein are kept. The tables are merged by keeping the target with the highest bit score (= highest scoring morpholog). The sequence of the morpholog is then retrieved from UniProt and used as a query for emapper. This finds the target sequence itself and retrieves the EggNOG entry, including preferred name and description.

Appendix B: Extracting quality indicators from the MorF workflow and comparing against physico-chemical parameters of proteins

The latest Foldseek version changes how results are ranked and e-values are calculated [112]. This change came too late in the manuscript revision process for us to repeat all analysis, but we encourage readers to use the latest version. This section refers to the version of Foldseek used in the manuscript (3-915ef7d) and may not be accurate any more.

To get a better understanding of the relationship between the various parameters of the MorF workflow as well as physico-chemical parameters of the proteins, we assessed the correlation between them (Fig. S2). We hoped to find a combination of measures that together would be a robust indicator of prediction quality for MorF. Additionally we sought to explore the relationship between structural prediction quality and various intrinsic protein parameters.

Most obviously, query length, (uncorrected) structural bit score, sequence bit score (emapper) as well as molecular weight correlate very strongly. Longer proteins (= heavier proteins) will have more or longer aligned regions with their targets, and as the bit score is additive, will accumulate higher bit scores, regardless of whether the alignment happens in structure or sequence space. It seems these measures can be used interchangeably; out of principle, we would prefer to use query length, as it is the least derived.

A way to overcome the dependence of bit scores on the length of the query protein is to normalise by the length of the aligned region (*corrected bit score (FS)*). This measure correlates with the percent structure state identity. This is equivalent to percent sequence identity correlating strongly with the bit score in a sequence similarity search, and is not surprising at all. More interestingly, these measures, together with the MSA size, correlate with the average pLDDT as a measure for protein prediction quality. Nevertheless, this correlation is less pronounced, suggesting to look at pLDDT independently.

Relative alignment length (FS) (percentage of the query (*Spongilla*) structure aligned with the best morpholog) only correlates weakly with other parameters.

Three things become apparent: first, higher pLDDT scores generally correlate with higher Foldseek bit scores. This means that Foldseek does a better job of identifying structural similarity for well-folded proteins. This seems intuitive: the atomic coordinates of well-folded proteins will be more deterministic than disordered proteins, so well-folded orthologs will overlap structurally over their entire length, while heavily disordered orthologs might only overlap structurally over very short patches. Additionally, high pLDDTs are reached when sufficiently large MSAs are available. Large MSAs contribute more information to AlphaFold's Evoformer module, leading to a higher predictive power.

Second, the relationship between pLDDT and (corrected) Foldseek bit score is not dependent on the length/weight of the protein. Independent of their size, proteins structures are predicted equally good or bad.

Third, pLDDT as well as Foldseek bit score do not correlate with various physico-chemical parameters of the proteins. Protein structure prediction seems to be independent from gravy score [116], aromaticity [115], isoelectric point, instability index

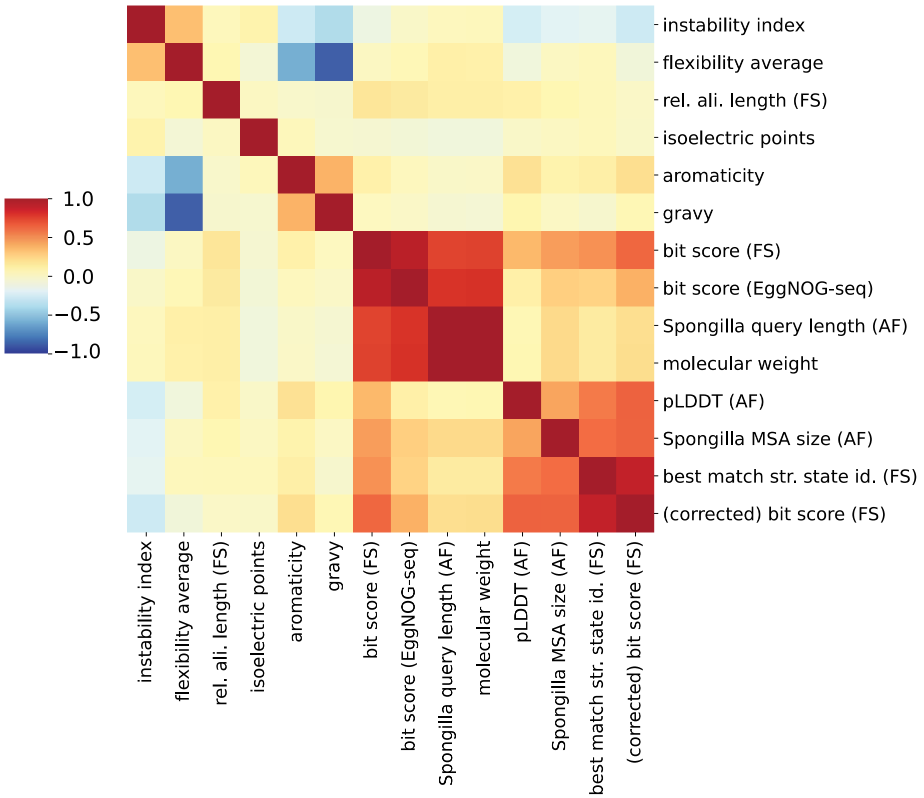

Figure S2 Pairwise correlation of metrics from the MorF workflow as well as physico-chemical protein parameters. *instability index*: measure for proteins stability based on Guruprasad et al. [113]. *flexibility average*: average of structural flexibility scores across all amino acids of a protein based on Vihinen et al. [114]. *rel. ali. length (FS)*: percentage of the query (*Spongilla*) structure aligned with the best target structure. *isoelectric points*: prediction of the isoelectric point of a protein. *aromaticity*: measure for protein aromaticity calculated as the relative frequency of Phe/Trp/Tyr [115]. *gravy*: measure for the hydropathic caracter of a protein based on [116]. *bit score (FS)*: bit score of the Foldseek alignment between the query *Spongilla* structure and the structure of the best Foldseek target. *bit score (EggNOG-seq)*: bit score of the EggNOG alignment between the query *Spongilla* peptide sequence and the best target sequence. *Spongilla query length (AF)*: the query length of the input peptide. *molecular weight*: molecular weight of the protein. *plDDT (AF)*: average pLDDT of the predicted structure. *Spongilla MSA size (AF)*: size of the input MSA used for AlphaFold. *best match seq. id. (FS)*: structure state identity in the alignment between the query. *corrected bit score (FS)*: the Foldseek bit score divided by alignment length.

[113] as well as the average of flexibility index [114] of proteins. Consequently, Foldseek bit score is not dependent on these parameters.

Next, we were interested in the distribution of these parameters across different annotation categories. Proteins either got an annotation by MorF and/or a sequence based emapper search or not. (*"Annotation"* can entail a name or a description of the protein). After applying the Foldseek bit score cut-off, we gain a total of 16,285 proteins with annotations from MorF and emapper, 15,599 proteins without annotations, 8,914 proteins with MorF annotations only and 1,147 proteins with sequence based annotations only (Fig. S3A). Comparing different MorF parameters, it becomes clear again that large MSAs are helpful for gaining MorF based annotations (Additional file 1: Fig. S3B). Nevertheless, MSAs are not strictly necessary for getting MorF based annotations. Comparing pLDDTs it becomes obvious that proteins

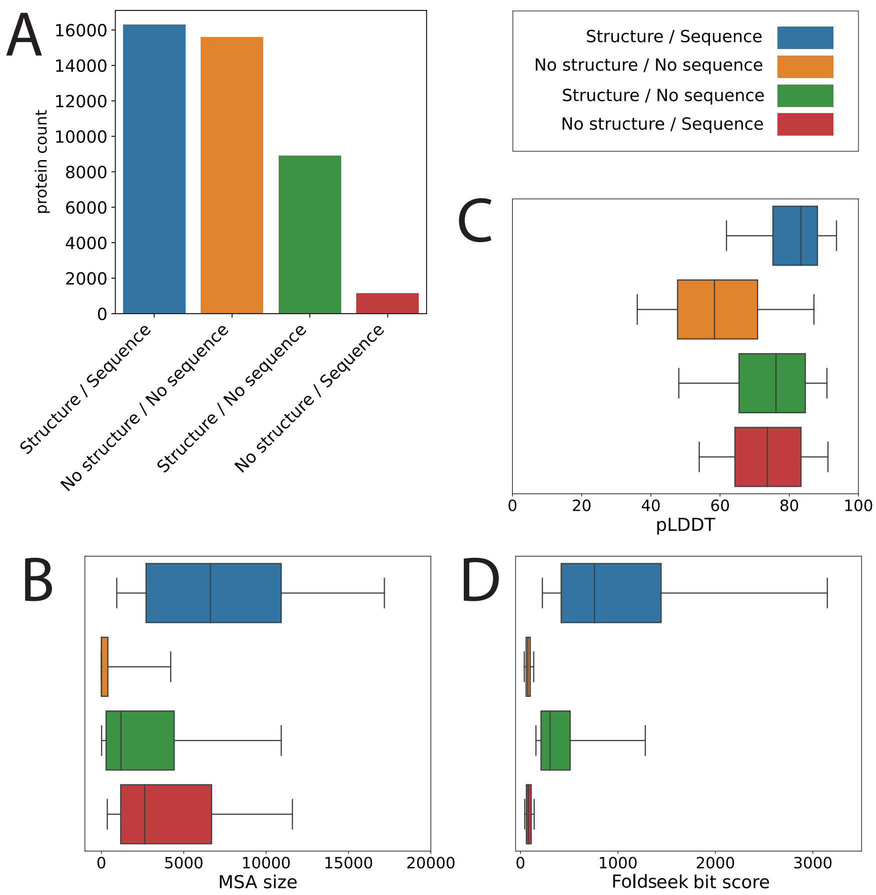

Figure S3 Comparison of MorF parameters in different annotation categories. A) Protein count in different annotation categories. Annotation includes either protein name or description. Protein counts are calculated *after* applying the Foldseek bit score cut-off. *"Structure/Sequence"*: Annotation by emapper sequence search as well as MorF *"No structure/No sequence"*: No annotation by emapper sequence search as well as MorF. *"Structure/No sequence"*: Annotation by MorF but no sequence based annotation by emapper. *"'No structure/Sequence"*: Annotation by emapper sequence search but not by MorF. B) Distribution of AlphaFold MSA sizes across annotation categories. C) Distribution of AlphaFold pLDDTs across annotation categories. D) Distribution of Foldseek (uncorrected) bit scores across annotation categories.

without any annotation usually have worse structural prediction quality (Fig. S3C) and vice versa. Interestingly, proteins with only sequence based annotation ("No structure / Sequence") have on average relatively high pLDDT values (Additional file 1: Fig. S3A). Nevertheless, their Foldseek bit score is too low (Additional file 1: Fig. S3D) to pass the bit score threshold for annotation. One possible explanation is that this category entails relatively short proteins (Additional file 1: Fig. S4A), which are shown to be suboptimal for structural searches [71], as they often do not pass (statistical) thresholds. On the other hand, proteins with MorF annotations have significantly higher Foldseek bit scores, reflecting the usefulness of the chosen bit score cut-off (Fig. S3D).

Comparing different annotation categories based on the distribution of physico-chemical parameters supports the statement above that they are not correlating

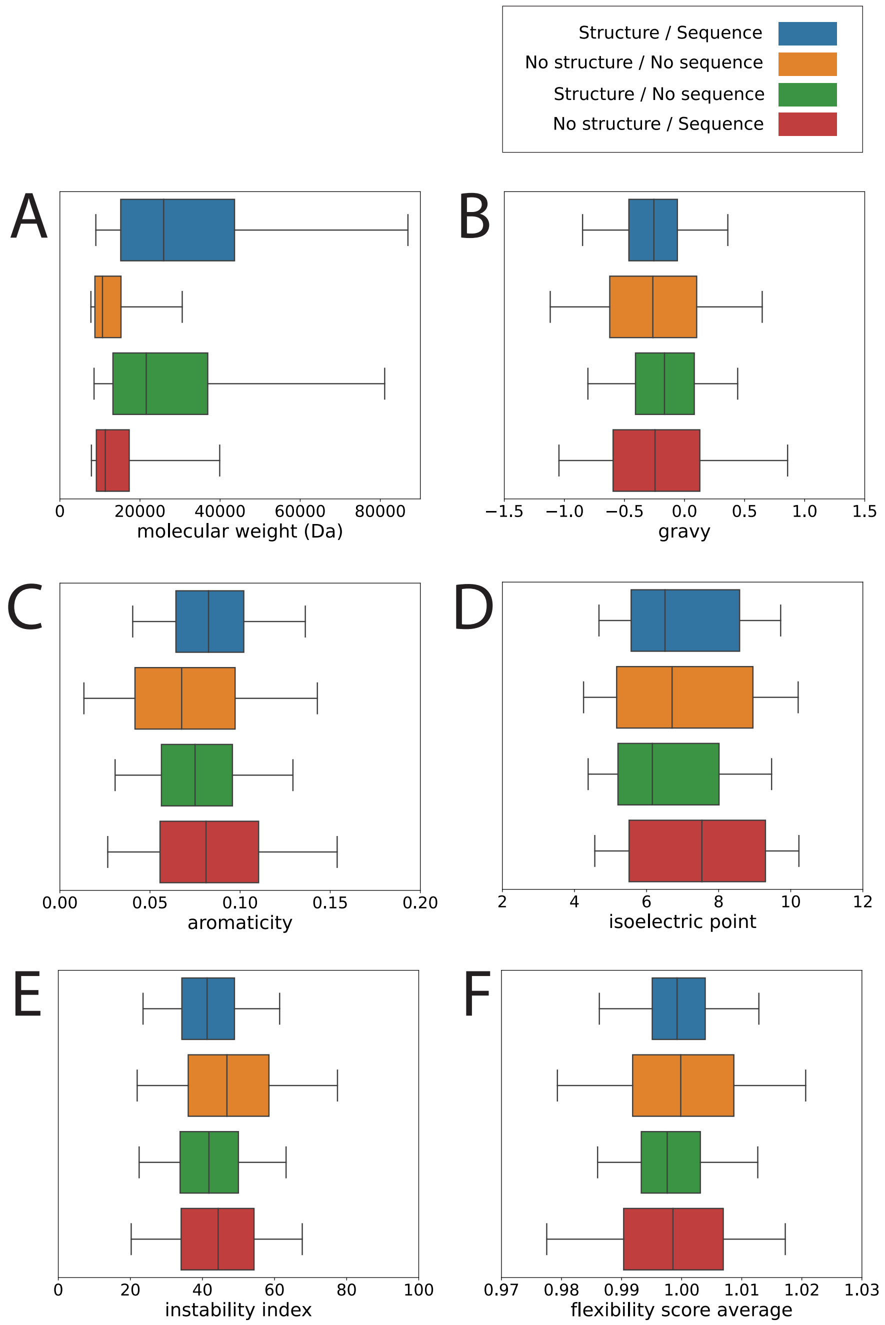

Figure S4 Comparison of physico-chemical protein parameters in different annotation categories. A) Distribution of molecular weights across annotation categories. B) Distribution of gravy scores across annotation categories. C) Distribution of aromaticity scores across annotation categories. D) Distribution of isoelectric points across annotation categories. E) Distribution of instability indices across annotation categories. F) Distribution of flexibility score averages across annotation categories.

with MorF parameters. Apart from molecular weight (Fig. S4B), none of the parameters such as gravy score, aromaticity index, isoelectric point, instability index and average flexibility score (Fig. S4B-F) differ significantly between the annotation categories.

Appendix C: Differentially expressed genes per cell type

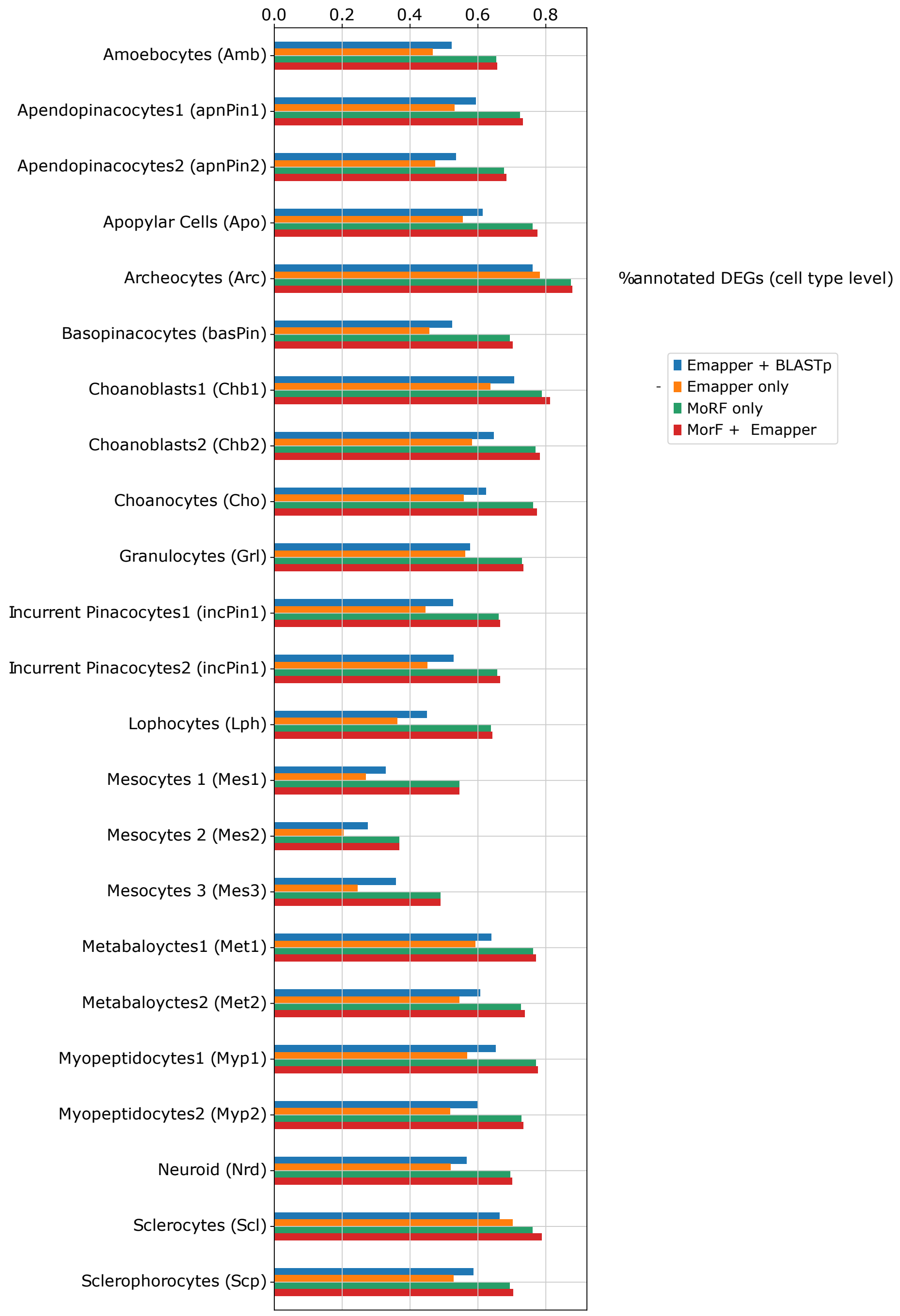

Figure S5 The percentage of marker genes per cluster as per Musser *et al.* that are annotated by different methods.

Appendix D: Comparison to sequence-profile searches

To gain a better idea of the limits of structural similarity as a means to transfer functional annotation we compared MorF's performance to that of HMMER [38]. In particular, we used eggnog-mapper in "HMMER" mode and performed sequence-to-profile searches for the translated *S. lacustris* proteome against the eukaryotic HMMs of the EggNOG database. Emapper-hmmer finds putative homologs for 28,897 proteins, compared to 17,990 by standard emapper and 25,232 morphologs found by by MorF (Fig. S6B, lower left).

The additional sensitivity of emapper-hmmer comes at the cost of precision, an expected trade-off, especially when using sequence profiles at the eukaryote level. Concretely, this means that we expect emapper-hmmer to return orthogroups from higher taxonomic levels more often than emapper or MorF, and therefore, in average, less orthogroups. It also means that we expect emapper-hmmer to fail to assign a name or a description to many annotations, as the taxonomic unit where homology is detected will be more vague than, e.g. a gene family. Since protein names, despite their drawbacks, are the most common human-readable short descriptors of function, this is a major drawback for the use of sequence profiles for functional annotation at scale, such as the use case we present here.

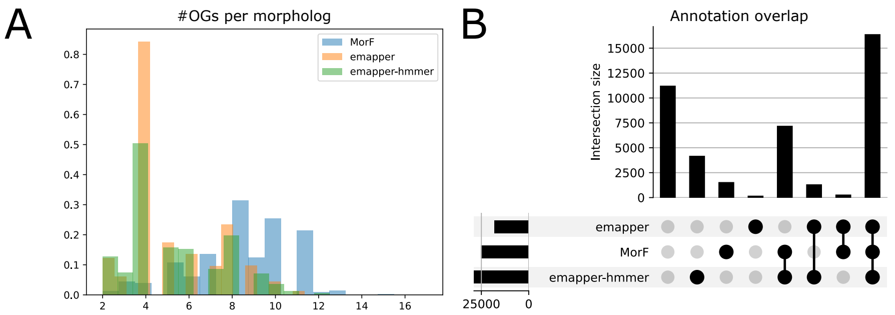

Figure S6 Comparison of MorF to sequence-based annotation A) Histogram of the number of EggNOG orthogroups assigned to each annotated query. As orthogroups have hierarchical relationships, more orthogroups generally indicate a more precise annotation. Mean and standard deviation: emapper (5.23, 2.08), emapper-hmmer (5.35, 2.23), MorF (8.28, 5.21). B) Breakdown of overlaps between different annotation strategies. The upper panel shows the number of *Spongilla* proteins annotated by emapper, MorF, emapper-hmmer, or their combinations. The lower right panel shows the categories being combined, and the lower left panel shows the total number of annotations produced by each tool.

Following our expectations, MorF results have significantly more orthogroups per query than either emapper (two-sample Kolmogorov-Smirnov test; statistic 0.56, p-value 0) or emapper-hmmer (two-sample Kolmogorov-Smirnov test; statistic 0.57, p-value 0) . Surprisingly, emapper-hmmer results have slightly more orthogroups per query than emapper, though with low statistical support (two-sample Kolmogorov-Smirnov test; statistic 0.057, p-value 6.9^{-24}) (Fig. S6A). In the same vein, 11,700 of the emapper-hmmer annotations don't have a preferred name, compared to 5,200-5,300 for MorF/standard emapper. Similarly, emapper-hmmer doesn't even produce a description for ca. 2,600 proteins, compared to 38 for MorF and ca. 600 for standard emapper.

tool combination	most specific OG	eukaryotic OG (eligible)	root OG
emapper / emapper-hmmer	66.74%	86.25% (16,032)	91.15%
MorF / emapper-hmmer	48.69%	82.06% (15,889)	87.18%
MorF / emapper	56.77%	89.11% (15,745)	90.71%

Table S1 Percentage of proteins where annotation agreed between the tools at different taxonomic levels. Where not explicitly noted, all 16,346 proteins were eligible for comparison.

At the same time, we examined the agreement between emapper-hmmer and MorF, similar to the comparison of MorF to emapper at the task of homology detection. For compatibility purposes, we focused on the 16,346 proteins that were annotated by all three methods (Fig. S6B). Since emapper-hmmer was run on the eukaryotic orthogroup profiles we added a comparison at the eukaryote orthogroup level. In the vast majority of cases MorF produces the same homology assignment as emapper-hmmer.

Furthermore we repeated the analysis presented in Fig. 1D, now including emapper-hmmer (Fig. S7). Reflecting the results that were already mentioned, emapper-hmmer performs better than standard emapper. However, when it comes to the marker genes that will drive biological discovery, MorF still outperforms sequence similarity as an annotation tool.

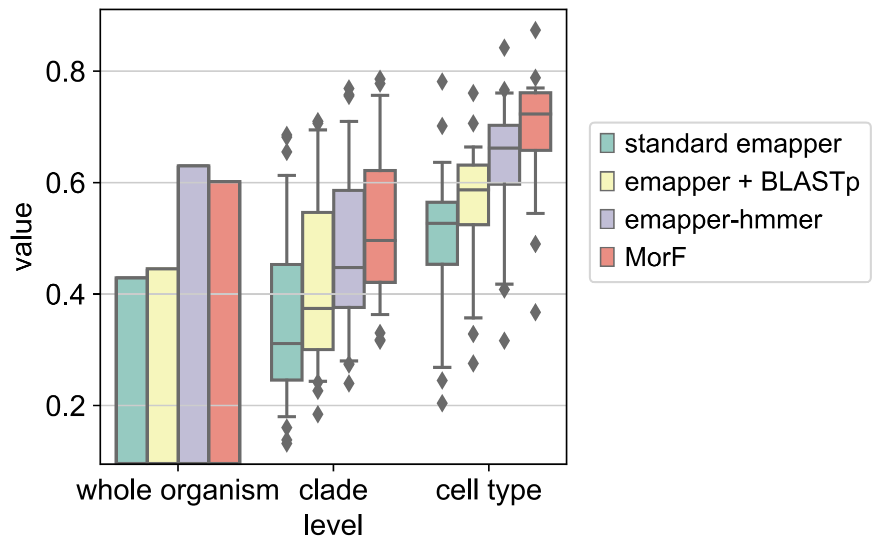

Figure S7 Proportion of annotated proteins using different annotation strategies. "Whole organism" denotes the entire proteome; "clade level" denotes the marker genes for cell type families in the single-cell data of Musser *et al.* [9], while "cell type" denotes the marker genes for the various cell types in the same dataset.

In conclusion, for the task of producing usable functional annotation, MorF compares favorably to emapper-hmmer, a state-of-the-art tool for profile-sequence searches. It proves capable of largely reproducing the same homology assignments, and annotates a higher proportion of cell type-specific genes, thereby aiding biological discovery.

Appendix E: GO term annotation comparison

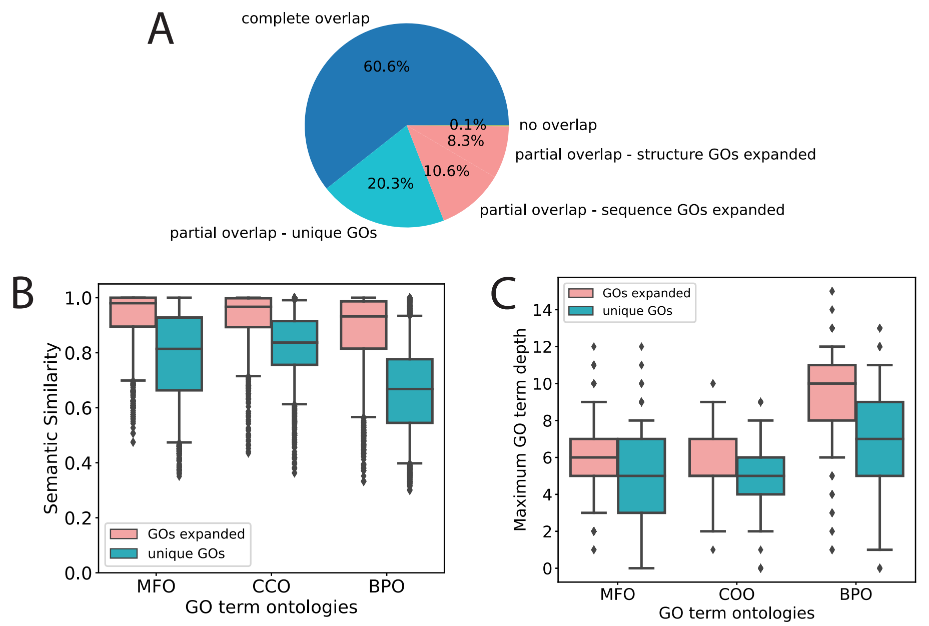

Figure S8 Comparison of GO term assignment, semantic similarity and depth between structure- and sequence based annotations A) Overlap of GO term assignments between MorF and sequence based annotations. ("complete overlap": GO term assignments are identical; "partial overlap - unique GOs": GO terms overlap with unique GO terms assigned to both, MorF and sequence based annotations; "partial overlap - sequence GOs expanded": additional GO terms assigned by sequence based annotation; "partial overlap - structure GOs expanded": additional GO terms assigned by MorF; "no overlap": no overlap between GO term assignments (17 cases total)) B) GO term semantic similarities between GO term assignments of the "partial overlap" categories across GO term ontologies ("MFO": molecular function, "CCO": cellular component, "BPO": biological process) C) Maximum depths of *overlapping* GO terms between sequence- and structure based annotations across GO term ontologies. Interpretation of GO term depths is challenging as different depths might imply different levels of details for different branches in the hierarchy.

isoform	q len	#MSA	%seq. id.	bit score	plddt	BLASTp
c88419_g1_i1	154	462	0.337	580.0	85.01	CRN5
c104256_g1_i1	375	163	0.272	785.0	82.42	CRN5
c104256_g1_i2	299	184	0.267	781.0	90.35	CRN5
c104256_g1_i3	336	167	0.262	777.0	88.39	CRN5
c104256_g1_i4	332	158	0.263	796.0	90.26	CRN5
c104256_g1_i6	253	196	0.275	774.0	92.81	CRN5
c94352_g2_i1	127	1,725	0.500	299.0	86.06	DD3-3
c89711_g1_i1	144	2,424	0.441	380.0	86.73	DD3-3
c89711_g2_i1	75	2,340	0.611	210.0	77.48	DD3-3
c112476_g1_i1	179	1,450	0.352	434.0	83.19	DD3-3
c103626_g1_i1	670	3,392	0.324	2224.0	91.33	DD3-3
c103626_g1_i2	670	3,424	0.329	2258.0	91.18	DD3-3
c103626_g2_i1	667	3,427	0.326	2215.0	90.01	DD3-3
c102584_g1_i1	578	3,328	0.355	2091.0	81.55	DD3-3
c102584_g1_i2	187	1,994	0.416	522.0	89.04	DD3-3
c101972_g1_i1	778	3,481	0.378	2536.0	85.58	DD3-3
c101972_g1_i2	778	3,481	0.378	2534.0	85.58	DD3-3
c101972_g1_i3	778	3,481	0.378	2537.0	85.54	DD3-3
c104453_g2_i1	359	368	0.177	662.0	75.80	foll. epith. yolk prot. subunit
c104453_g2_i2	280	356	0.172	658.0	89.90	foll. epith. yolk prot. subunit
c104453_g2_i3	340	378	0.185	634.0	78.61	foll. epith. yolk prot. subunit
c104453_g2_i4	236	374	0.183	594.0	83.73	foll. epith. yolk prot. subunit
c104453_g3_i1	182	341	0.117	298.0	70.54	foll. epith. yolk prot. subunit
c98985_g1_i2	73	1,852	0.138	45.0	70.12	XPO5 (Exportin-5)
c88005_g3_i1	130	3,213	0.274	363.0	94.66	hemopexin repeat-containing protein
c70965_g1_i1	169	299	0.209	354.0	77.62	pinocchio
c103612_g1_i1	89	1,253	0.152	46.0	73.88	kinesin-like protein KIF23
c102197_g1_i3	312	257	0.179	649.0	86.28	bamB
c100331_g2_i1	281	43,509	0.219	821.0	82.62	hydralysin-2-like
c93555_g2_i2	77	439	0.109	80.0	87.77	?
c86044_g1_i1	87	7,052	0.077	90.0	88.87	?
c83472_g1_i1	98	553	0.177	90.0	84.77	?
c108675_g1_i1	73	3,174	0.224	51.0	78.17	?
c100701_g1_i2	69	1	0.123	61.0	81.77	?
c100429_g1_i1	79	43,996	0.152	85.0	71.74	?

Table S2 High confidence structures with non-helical appearance. The last column contains a summary of the significant BLASTp results in the `nr` database. Question marks denote peptides who did not find any named sequence homologs or significant PFAM domain hits.

Appendix F: Mesocyte marker gene expression

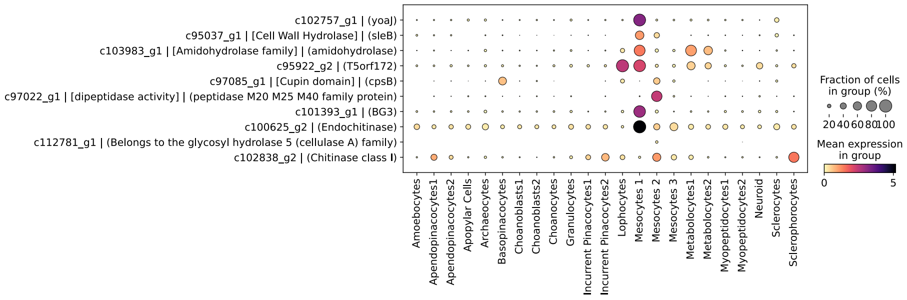

Figure S9 Mesocyte marker gene expression Dotplot of mesocyte marker gene expression for HGT candidates. emapper annotation in square brackets; MorF annotation in round brackets. Mean expression of log-transformed, normalised counts.

Appendix G: Comparison of MorF parameters between different agreement categories of structure and sequence based annotations

In order to explore and explain disagreements between structure and sequence based annotations, we compared different MorF parameters such as query length, MSA size, structure prediction quality as well as (alignment length) corrected Foldseek bit scores across different annotation agreement categories from (Fig. 1C; also refer to the corresponding notebook [16]). Most proteins do not have sequence (EggNOG) based annotations (Fig. S10A) and are excluded from this anaysis. This comparison shows that MSA sizes and corrected bit scores (Fig. S10E-F) are lower for proteins in the "no agreement" category compared to proteins with (partially) overlapping sequence and structure annotations. At the same time, the structure prediction quality for proteins (pLDDT) in the "no agreement" category are only marginally lower (Fig. S10C). Comparing Foldseek corrected bit scores across the annotation agreement categories broken down to multiple pLDDT buckets (Fig. S10B) shows that even in the cases of high prediction quality ($pLDDT = 90 - 95\%$) the corrected Foldseek bit scores for the "no agreement" cases are very low, potentially leading to uncertainty in the structurally guided functional annotation. There are two potential reasons for this observation: First, Foldseek search results depend on the content of the structural databases that Foldseek searches against. Even very good structural predictions of sponge proteins can have low Foldseek bit scores if there are no morphologs present in the Foldseek database. With the ever expanding prediction of structures in the AlphaFold database, this issue will possibly be mitigated in the future. In fact, during the review process of this manuscript, a new version of AlphaFoldDB has been released, containing protein structures from all UniProt entries. Second, comparing query lengths of proteins in different annotation agreement levels (Fig. S10D), we observed a large proportion of long proteins in the "no agreement" category. Longer proteins often are composed of multiple domains that are linked via flexible linkers. Manual inspection showed that ColabFold correctly predicts the (globular) domains, leading to overall high pLDDT values, while predicting the flexible linker seemingly in a random fashion. This in turn leads to a random positioning of the domains relative to each other. This poses an issue for the subsequent Foldseek search in which only one of the domains can be superposed correctly, leading to the observed low corrected bit score. However, the extent to which this leads to incorrect functional annotation is still unknown and is outside the scope of the study.

Taking the possible explanations into account, sequence annotation would for now be preferable for proteins in the "no agreement" category. This agrees with our approach in the manuscript in which we primarily give preference to "legacy" sequence based annotation while supporting them with structural predictions and expand the annotation of those cases in which sequence is not sufficient for functional annotation.

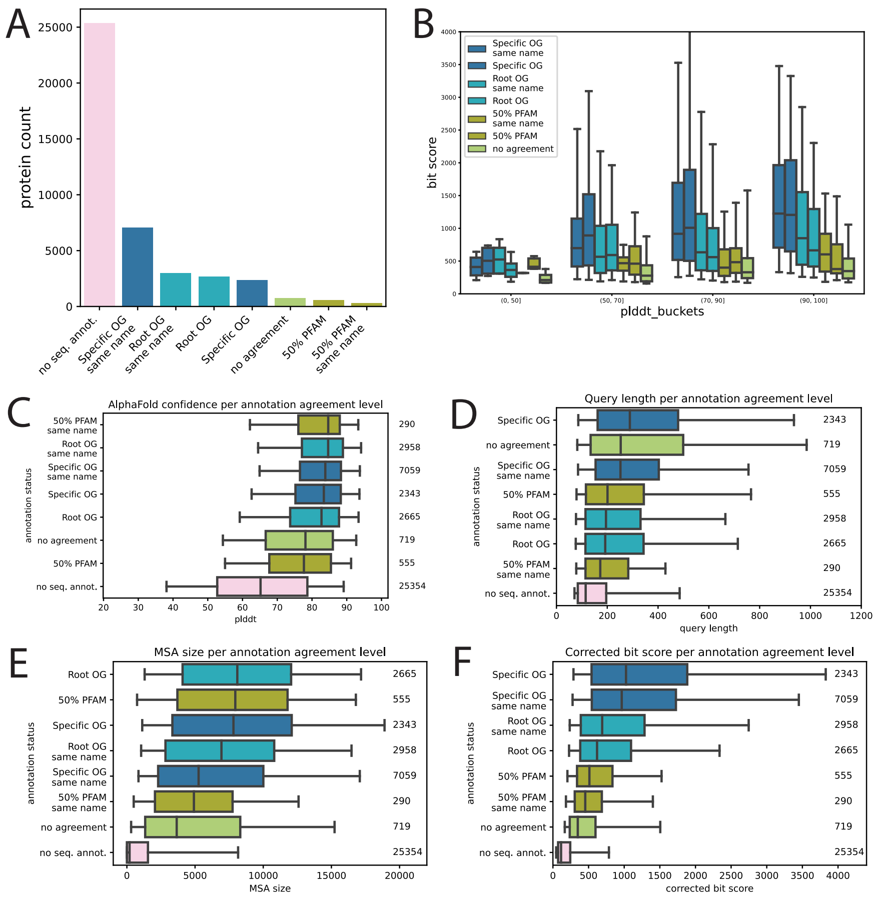

Figure S10 Comparison of MorF parameters between different categories of structure and sequence annotation agreements A) Protein count of different annotation agreement categories. Most proteins ($\sim 25,000$) do not have sequence (emapper) based annotations. 719 proteins do not agree in their sequence and MorF based annotations. B) Corrected (Foldseek) bit score comparisons across different annotation agreement categories within pLDDT value buckets. In all pLDDT buckets, proteins in the "no agreement" category show lowest average Foldseek bit scores. Structure prediction quality (pLDDT) (C), query length (D), MSA size (E) and corrected bit score (F) comparisons across annotation agreement categories.

Appendix H: Functional similarity of top morphologs

An indirect way to assess the performance of MorF at transferring functional annotations is to check how consistenly the top morphologs are assigned the same function. To do that, we define top morphologs via query-specific thresholds [117] and then compare the EC numbers of the top results to the best result [118], which in MorF would be assigned to the query protein.

We examined the AlphaFoldDB morphologs for each *Spongilla* query and calculated two thresholds to define the best morphologs. One is the multi-Otsu threshold [119], a technique from image analysis that tries to maximise between-class variance when thresholding pixel intensity values. The second threshold, referred to as "90th percentile", was defined as the 90th percentile of the bit score range of each query's putative morphologs. The 90th percentile threshold was much stricter than the multi-Otsu threshold in all cases.

We thresholded the AlphaFoldDB result matrix with the multi-Otsu cutoff for each query and queried UniProt via the Proteins API [120] to get the sequences of each protein in the filtered matrix. We submitted the sequences to the emapper webserver and removed hits with an e-value worse than 10^{100} to make sure only self-matches were kept. We populated the AlphaFoldDB result matrix with the EggNOG annotation and removed entries that didn't have an EC number. We then additionally removed all queries that only had one possible target remaining.

We then assessed the functional overlap of the Otsu-thresholded and 90th percentile-thresholded morphologs with the top morpholog. We quantified this as the average agreement of the EC numbers of the morphologs with the EC number of the top morpholog. An average agreement of 3 would mean that the top morphologs, on average, share the first three positions of the EC number of the top morpholog.

A total of 24,380 queries had at least two morphologs with an EC clearing the multi-Otsu threshold, with an average of 13.74 top morphologs per query. A total of 7,072 queries had at least two morphologs with an EC clearing the much stricter 90th percentile threshold, with an average of 7.37 top morphologs per query.

The Otsu threshold seemed to be too permissive, with a large majority of the queries showing significant divergence within their top morphologs (average EC overlap of 2.02, so barely within the same enzyme subclass). On the other hand, in the 90th percentile threshold top morphologs had an average EC overlap of 3.7 - close to complete agreement. In the same subset of proteins (90th percentile threshold), the first and second best morphologs have a different EC in 10.88% of cases.

For the narrow subset of proteins we could feasibly examine, we conclude that the top morphologs of each protein overwhelmingly have the same function, offering another indication that MorF's approach is fundamentally valid.

Appendix I: Structure-sequence agreement in model species

As proof of principle we explored the power of structural similarity to identify homologous genes for a number of model species whose proteomes already had predicted structures in AlphaFoldDB. We aligned AlphaFoldDB against itself using Foldseek. For each query protein we kept the best target from outside the taxonomic group and compared the eukaryotic orthogroup assignment. The best structural hit overwhelmingly belonged to the same orthogroup as the query (Additional file 1: Table S3).

We used the Proteins API [120] to obtain the species name for each entry [121]. We decorated each entry with its orthogroup assignment from the EggNOG database [18] (v5.0). Not all proteins currently in AlphaFoldDB could be assigned an orthogroup; we ignored comparisons where the query or its best target were missing the orthogroup assignment. The full table can be found in the corresponding notebook [122].

We compared the remaining eligible cases and found overwhelming agreement between the eukaryotic orthogroup of the best structural target and the orthogroup of the query protein, confirming that structural similarity can, in principle, detect homology relationships. Though the available targets were restricted to be outside the clade, order, phylum, or even kingdom, top morphologs still are homologs in the large majority of cases, a very encouraging indication that MorF is able to detect homology over long evolutionary distances.

As a more demanding application, we compared enzyme function between distantly related species to see how often function was correctly predicted by structural similarity (see corr. notebook [123]). In particular, we were interested in cases where homology via sequence similarity was no longer detectable but the EC number still overlapped.

We performed two comparisons between well-studied, distantly related eukaryote species: baker's yeast against human, and *Arabidopsis thaliana* against human. We downloaded the predicted protein structures from the AlphaFold protein database, performed structural alignment against AlphaFoldDB, and then filtered out non-human hits. Using the EggNOG annotation of the query and target proteins, we removed pairs that belonged to the same protein families (same root orthogroup or same most specific orthogroup; opisthokont for human/yeast and eukaryote for *A. thaliana*.)

The best morphologs of yeast enzymes without previously detected homologs in human agree on all four positions of the EC number in 53/146 (36%) cases. Broader similarity (three of four EC positions) can be found in 109/146 (75%) cases. Similarly, the best morphologs of *A. thaliana* enzymes without clear homologs in human have the same EC in 176/532 (33%) cases, and share the first three digits in 357/532 (67%) cases.

species	#queries with out-of-group targets	#eligible queries	%eligible agreement	taxonomic group
H. sapiens	20,392	13,838	74.30%	Vertebrata
M. musculus	21,507	15,084	75.05%	Vertebrata
R. norvegicus	19,246	13,180	74.60%	Vertebrata
D. rerio	24,626	13,303	75.36%	Vertebrata
D. melanogaster	13,418	8,828	79.69%	Arthropoda
C. elegans	19,599	11,177	67.29%	Nematoda
S. mansoni	13,845	1,730	91.85%	Nematoda
A. thaliana	27,357	19,800	83.78%	Eudicots
G. max	55,759	32,239	85.40%	Eudicots
Z. mays	38,818	18,644	85.47%	Monocots
O. sativa subsp. japonica	41,794	20,306	86.51%	Monocots
S. cerevisiae (str. ATCC 204508/S288c)	6,013	4,347	83.41%	Saccharomycetales
S. pombe (str. 972/ ATCC 24843)	5,104	3,982	90.51%	Saccharomycetales
C. albicans (str. SC5314/ ATCC MYA-2876)	5,973	4,118	84.41%	Saccharomycetales
C. carrionii	11,169	7,040	81.29%	Chaetothyriales
P. lutzii (str. ATCC MYA-826/Pb01)	8,791	5,086	84.19%	Eurotiomycetes
S. schenckii (str. ATCC 58,251/ de Perez 2211183)	8,652	6,349	78.41%	Sordariomycetes
T. brucei brucei (str. 927/4 GUTat10.1)	8,476	4,303	60.26%	Trypanosomatida
T. cruzi (str. CL Brener)	19,026	8,782	56.56%	Trypanosomatida
L. infantum	7,914	4,423	56.14%	Trypanosomatida
D. discoideum	12,584	6,312	63.96%	Amoebozoa

Table S3 Structural similarity correctly identifies homologs for model species in the absence of close relatives. Columns: total number of queries with a hit outside the species context; total number of cases where both query and best non-species target are annotated by EggNOG; percentage of these cases where the eukaryote orthogroup between query and target is the same; taxonomic group. Species that belong to the same taxonomic group were excluded when performing this comparison.

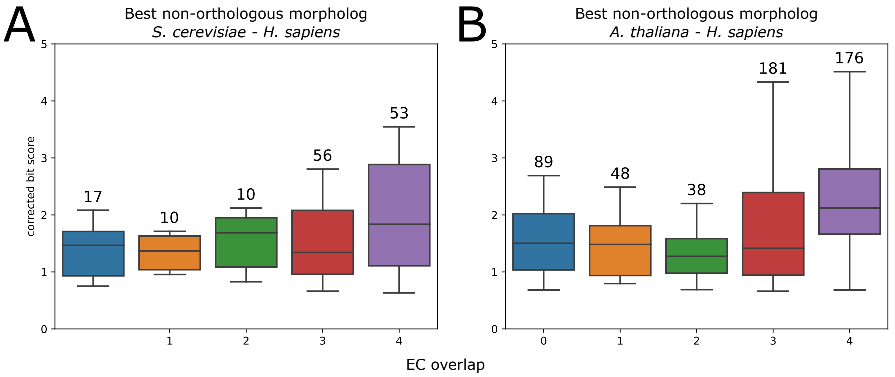

Figure S11 Remote species comparison: Distribution of the EC overlap of the best non-homologous morphologs between distantly related species, against corrected bit score. For the majority of cases, same structure means a significant overlap in EC despite the absence of homology. While high EC overlap usually means a higher corrected bit score (bit score divided by alignment length), it is not always the case.

gene ID	MorF annotation	blastp result	blastp e-value/seq id/ query coverage	EggNOG 5.0 result	EggNOG e-value	Comment
c105746_g1	CopC	-	-	-	-	MorF
c87345_g1	ApoM	-	-	-	-	MorF
c95564_g1	SRXN1	-	-	-	-	MorF
c103725_g3	FLC2/3	-	-	-	-	MorF
c100824_g2	DsbB	-	-	-	-	MorF
c103888_g2	FLC3	-	-	-	-	MorF
c100917_g2	APEX1	-	-	Endo-/Exonuclease phosphatase	3.60E-08	Specified by MorF
c99704_g1	HPGDS	GST	5E-46/33%/97%	GST	3.10E-32	Specified by MorF
c104290_g2	NmrA-like	-	-	NADPH binding	2.90E-98	Specified by MorF

Table S4 Blastp and EggNOG search results of newly identified myopeptidocyte marker gene morphologs. Blastp searches were run against non redundant protein database (nr) in default mode. EggNOG sequence searches were run in default mode. E-value, sequence identity (seq id) and query coverage are reported from the best blastp hit or EggNOG orthologous group.

Appendix J: Myopeptidocyte marker genes

gene ID	cell type	putative function	blastp nr - taxonomic profil	blastp nr e-value/seq id/ query coverage	blastp nr_env	blastp nr_env e-value/seq id/ query coverage	EggNOG 5.0 result	EggNOG e-value	MorF annotation	annotation source	Gene origin	putative HGT event timing
c97022_g1	Mes 1	proteinase	argE/peptidase M20 - A. queenslandica/ Streptophyta/Bacteria	5E-155/51%/97%	hypothetical protein - marine metagenome	1E-104/45%/87%	Peptidase M20A family - Proteobacteria	2E-34	Metallopeptidase M20 family	legacy	Likely bacterial origin	500 mya
c103983_g1	Mes 1	hydrolase acting on aminogroups	formamid deformylase/ aminohydrolase - Cnidaria/Porifera/Bacteria	5E-144/42%/96%	formamid deformylase - subsurface/marine metagenome	5E-99 / 35% / 96%	metal-dependent hydrolase - Proteobacteria	5.90E-130	Aminohydrolase	legacy	Likely bacterial origin	500 mya
c102757_g1	Mes 1	cell wall degradation	uncharacterised protein - Rotaria/Daphnia	1E-101/50%/90%	-	-	unknown function - Daphnia	1.70E-85	yoaJ	refined by MorF	Likely Metazoan origin	15-300 mya
c95922_g2	Mes 1	hydrolase activity	hypothetical protein - Metazoa/Fungi/Bacteria	3E-14/41%/66%	hypothetical protein - marine metagenome	9E-08/40%/46%	T5orf172 - Bacteria	3E-09	T5orf172	MorF	Unresolved	15-300 mya
c101393_g1	Mes 1	cellulase activity	hypothetical protein - Proteobacteria	9E-130/56%/66%	hypothetical protein - compost metagenome	4E-40/34%/65%	unknown function - Bacteria	4.50E-52	BG3	MorF	Likely bacterial origin	15-300 mya
c100625_g2	Mes 1	endochitinase	hypothetical protein - Fungi	1E-13/69%/43%	-	-	chitin-binding - Eukaryota/Streptophyta	2.50E-13	Endochitinase	MorF	Likely eukaryotic origin	15-300 mya
c95037_g1	Mes 1/2	hydrolase activity cell wall organisation	cell wall hydrolase - Alphaproteobacteria/ Planctomycetales/etc.	3E-45/56%/94%	cell wall hydrolase - marine/compost metagenome	4E-38 / 51% / 94%	sporulation - Bacteria	2.80E-24	sleB	refined by MorF	Likely Alphaproteobacteria origin	15-300 mya
c97085_g1	Mes 2	xanthan biosynthesis	cupin-domain containing protein - CFB group bacteria	6E-45/62%/47%	hypothetical protein - viral metagenome	7E-101/60%/96%	Mannose-6-phosphate isomerase - Proteobacteria	1.10E-19	GMHA	legacy	Likely bacterial origin	15-300 mya
c112781_g1	Mes 2	cellulase activity	hypothetical protein - CFB group bacteria	4E-24/48%/100%	-	-	unknown function - Bacteria	7.30E-29	Cellulose A family member	MorF	Likely CFB group bacteria origin	15-300 mya
c102838_g2	Mes 2	endochitinase	unnamed product - Fungi/Dinoflagellata	1E-09/47%/33%	-	-	chitin-binding - Eukaryota/Streptophyta	9E-11	Chitinase class I	new annotation	Likely eukaryotic origin	15-300 mya

Table S5 Blastp and EggNOG search results of newly identified mesocyte marker gene morphologs. Blastp searches were run against non redundant protein database (nr) as well as environmental metagenome databases (env_nr) in default mode. EggNOG sequence searches were run in default mode. E-value, sequence identity (seq id) and query coverage are reported from the best blastp hits or best scoring EggNOG orthologous group. Based on the blastp and EggNOG results we hypothesised plausible gene origins. Potential timing of putative HGT events are based on the distribution of the genes within Demosponge phylogeny [124].

Appendix K: Mesocyte marker genes

Appendix L: Presence of sponge HGT candidates in Choanoflagellates

To gain a more complete picture about the putative HGT events suggested by MorF we performed sensitive sequence searches (MMseqs2, setting `-s 7.0`; see [125]) of the *Spongilla* HGT candidates against the refernce proteomes of emerging Choanoflagellate models *S. rosetta* and *M. brevicollis*. We find *Spongilla* genes c103983_g1 and c97022_g1 in both species, with rather low e-values. This is intriguing, as all the *S. rosetta* targets had already been identified as putative HGT results in earlier work [64]; in conjunction with their broad phylogenetic distribution in sponges it is tempting to speculate that these are ancient HGT events that occurred before the split of Choanoflagellates and sponges.
